# The Country Profiles of the PHARMINE Survey of European Higher Educational Institutions Delivering Pharmacy Education and Training

**DOI:** 10.3390/pharmacy5030034

**Published:** 2017-06-22

**Authors:** Jeffrey Atkinson

**Affiliations:** Pharmacolor Consultants Nancy, Villers 54600, France; jeffrey.atkinson@univ-lorraine.fr

**Keywords:** pharmacy, education, practice, Europe

## Abstract

The PHARMINE (Pharmacy Education in Europe) consortium surveyed pharmacy education and practice in 2012. Surveys were updated in 2017 for publication. The PHARMINE consortium was especially interested in specialization in pharmacy education and practice (for community, hospital, and industrial pharmacy), and in the impact of the Bologna agreement and the directive of the European Commission on education and training for the sectoral profession of pharmacy on European degree courses. The surveys underline the varying attitudes of the different European countries to these various aspects. The surveys will now be published in *Pharmacy*. They will be useful to researchers in education, and to staff and students interested in mobility amongst different European and/or non-European countries. In order to assure a full understanding of the country profiles to be published in the journal *Pharmacy*, this introductory article describes the general format of the survey questionnaire used.

## 1. Introduction

In the 21st century, the role of pharmacists is changing. For instance, community and hospital pharmacists play an increasingly important role individually (monitoring of chronic diseases, vaccinations, etc.), and as partners, in the efficient use of health care resources [1]. Industrial pharmacists are major players in the developmental transition of the pharmaceutical industry [2] as it undergoes a transformation from a chemical, small molecule industry to a biotechnological, peptide industry. For all pharmacists, such new functions will require new competences.

The PHAR-QA (Quality Assurance in Pharmacy Education and Training) [3] consortium investigated such competences. However, before proposing a framework of new competences, it was necessary to survey the present state of pharmacy education and training (PET) in Europe in order to establish capacity in European PET. This was done by the PHARMINE (Pharmacy Education in Europe) consortium [4] which prefigured the PHAR-QA consortium.

The PHARMINE project brought together the academic members of the European Association of Faculties of Pharmacy (EAFP) [5], and EU partner associations representing pharmaceutical practitioners: community (PGEU) [6], hospital (EAHP) [7], and industrial pharmacy (EIPG) [8]. The European Pharmacy Students’ Association [9] was also an important partner.

The PHARMINE consortium produced profiles for the countries in the European Higher Education Area [10] by surveying pharmacy practice and resources, management, and curricula of pharmacy degree courses. Resources and curricula were surveyed at the fundamental, general practice level and at the advanced specialization level (i.e., community, hospital, and industrial pharmacy elective courses).

Finally, the consortium examined the opportunities in different countries for the introduction into PET of the principles of the Bologna declaration [11] on European university degree courses (not only pharmacy) including the potential separation of a tunnel-structured five-year degree course such as that for pharmacy, into a basic three-year bachelor course followed by an advanced five-year master course. It should be noted that the Bologna declaration, and its subsequent amendments, are the fruit of the collaboration of the ministers of education of the different European countries. This does not represent European Union (EU) law.

In parallel, the consortium examined to what extent different countries abide by the proposals in the European Commission’s (EC) directive on education and training for the sectoral professions such as pharmacy, concerning management of the degree course (pharmacy being a five-year tunnel course in the eyes of the EC) and subject area content of the course [12]. Subject area content will have a direct impact on the competences to be taught. It should be noted that an EC directive is an instrument of the law of the European Union, and as such is to be adopted by the different member states into their national legislation.

## 2. Methodology

The PHARMINE questionnaire was based on that used in a first survey of pharmacy departments carried out by the EAFP in the 1990s [13].

The project ran from spring 2009 through summer 2011. All country profiles were updated in the spring of 2017 for publication.

The survey form was sent out to at least one department in each country of the European Higher Education Area. Data for the survey were collected by electronic means [14]. This was backed up by telephone calls and/or by on-site visits to help and encourage respondents to fill out the form.

## 3. Results and Discussion

### 3.1. Survey Chapters

The survey document had eight chapters:

1. Personal details of respondent
2. Organization of the activities of pharmacists, professional bodies
3. Pharmacy higher education institutions (HEIs), students, and courses
4. Teaching and learning methods
5. Subject areas
6. Impact of the Bologna principles
7. Impact of EC directive
8. Quality assurance or pharmacy education and training (E&T)

### 3.2. Personal Details of the HEI Respondent

The survey asked for contact details (name, address, telephone, email, and website) of at least one person from each HEI. The contact person implicitly agreed to act as contact for enquires from students, staff, researchers, and/or practitioners once the survey had been published.

PHARMINE also asked respondents to provide useful website addresses (for mobility…) and supporting material such as texts of national law.

Respondents were then asked to give information on the following chapters.

### 3.3. Organization of the Activities of Pharmacists, Professional Bodies

The aim was to produce sufficient data (numbers, descriptions…) to draw a realistic and relevant picture of the practice of community, hospital, industrial pharmacists, and pharmacists working in other professions, as well as pharmacy technicians. A final section dealt with the role of the professional bodies in legal and ethical matters.

Questions were asked on the following topics.

Pharmacy practice

1. Community pharmacy practice
  - Numbers of community pharmacies and pharmacists
  - Competences and roles of community pharmacists
  - Whether the ownership of community pharmacies is limited to pharmacists
  - The rules governing the geographical distribution of community pharmacies
  - The availability of drugs and other healthcare products by channels other than pharmacies
2. Pharmacy technicians
  - Their titles and number(s)
  - Their qualifications
    - Organizations providing and validating their E&T
    - Duration of studies
    - Subject areas
  - Their competences and roles
3. Hospital pharmacy practice
  - Whether such a function exists. PHARMINE was interested in whether “hospital pharmacy practice” could be defined by work place or duties, i.e., is a hospital pharmacist a pharmacist who works in a hospital or a pharmacist with specific, legally defined tasks related to practice with hospitalized patients?
  - Number of hospital pharmacists
  - Number of hospital pharmacies
4. Industrial pharmacy
  - Pharmaceutical and related industries
    - Number of companies with production, R&D, and/or distribution
    - Number of companies producing generic drugs only
  - Industrial pharmacists
    - Number of pharmacists working in industry
    - Competences and roles of industrial pharmacists
5. Pharmacists working in other sectors
  - Number of pharmacists working in other sectors
  - Sectors in which pharmacists are employed
  - Competences and roles of pharmacists employed in other sectors

Professional associations: roles.

1. Registration of pharmacists
2. Creation of community pharmacies and control of territorial distribution
3. Ethics and professional conduct
4. Quality assurance and validation of HEI courses for pharmacists

In this and other sections numerical data for individual countries was compared to those for Europe [15].

### 3.4. Pharmacy HEIs, Students, and Courses

The aim was to produce sufficient data (numbers, descriptions…) to draw a realistic and relevant picture of PET. A final section concerned the past and future changes in PET.

Questions were asked on the following topics.

1. HEIs
  - Total number of pharmacy HEIs in your country, public and private
  - Independent department or attached to a science or medical faculty
  - B + M degree structure
    - Availability
    - M open to students with a non-pharmacy B degree
2. Staff (at the level of the country and at that of the responding HEI)
  - Number of teaching staff (nationals)
  - Number of international teaching staff—European and non-European
  - Number professionals (pharmacists and others) from outside the HEI, involved in pharmacy E&T
3. Students (at the level of the country and at that of the responding HEI)
  - Number of places at traditional entry (beginning of S1/B1, following secondary school)
  - Number of applicants for entry
  - Number of graduates that become professional pharmacists.
  - Number of international students—European (Erasmus) [16] and non-European
  - Specific pharmacy-related (national or HEI) entrance examination
  - National numerus clausus
  - Advanced entry (>S1/B1)
    - Which level
    - Requirements
  - Fees for home, European, and non-European students
4. Specialization
  - Which years
  - Which topics (industry, hospital…)
  - Student numbers in each specialization
5. Changes in PET: past and future

### 3.5. Teaching and Learning Methods

The aim was to produce sufficient data (numbers, descriptions…) to draw a realistic and relevant picture of the methods used in teaching and learning.

Questions were asked on the following topics.

1. Course organization
  - Student hours in each year for lectures, tutorials, practicums, independent project work
  - Traineeship (community, hospital, industry)
  - Electives
  - Validation of courses, etc.

### 3.6. Subject Areas

The aim was to produce sufficient data (numbers, descriptions…) to draw a realistic and relevant picture of the methods used in teaching and learning.

Questions were asked on the number of hours per year in each of the following seven subject areas (slightly modified version of definition of subject areas used in first EAFP survey cited above).

1. Chemical sciences (CHEMSCI)
  - General, organic, and inorganic chemistry
  - Analytical chemistry
  - Pharmaceutical chemistry and pharmacopeial analysis
  - Medicinal physico-chemistry/structure-activity/drug design
2. Physical and mathematical sciences (PHYSMATH)
  - Physics
  - Mathematics, pharmaceutical calculations
  - Information technology, information technology applied to community pharmacy, information technology applied to national health-care
  - Statistics
  - Experimental design and analysis
3. Biological sciences (BIOLSCI)
  - Foundation biology
  - Cell biology
  - Botany
  - Mycology
  - Zoology
  - Biochemistry
  - Molecular biology
  - Genetics
4. Pharmaceutical technology (PHARMTECH)
  - Formulation
  - Drug disposition and metabolism/pharmacokinetics
  - Novel drug delivery systems
  - Drug design
  - Pharmaceutical R&D
  - Drug production
  - Quality assurance in production
  - Drug/new chemical entity registration and regularization
  - Common technical document (quality (pharmaceutical), safety (safety pharmacology and toxicology), efficacy (preclinical and clinical studies))
  - Ophthalmic preparations
  - Medical gases
  - Cosmetics
  - Management strategy in industry
  - Economics of the pharmaceutical industry and R&D
5. Medicinal sciences (MEDISCI)
  - Human anatomy and physiology
  - Medical terminology
  - Pharmacology
  - Pharmacognosy
  - Pharmacotherapy/therapeutics
  - Toxicology
  - Pathology, histology
  - Microbiology
  - Nutrition, non-pharmacological treatment
  - Hematology
  - Immunology
  - Parasitology
  - Hygiene
  - Emergency therapy
  - Clinical chemistry/bioanalysis (of body fluids)
  - Radiochemistry
  - Dispensing process, drug prescription, prescription analysis (detection of adverse effects and drug interactions)
  - Generic drugs
  - Planning, running, and interpretation of the data of clinical trials
  - Medical devices
  - Orthopedics
  - OTC medicines, complementary therapy
  - At-home support and care
  - Skin illness and treatment
  - Homeopathy
  - Phytotherapy
  aa. Drugs in veterinary medicine
  bb. Pharmaceutical care, pharmaceutical therapy of illness and disease
6. Law and social sciences (LAWSOC)
  - Legislation, law relating to pharmacy
  - Social sciences
  - Forensic science
  - Professional ethics
  - Philosophy
  - Economics, financial affairs, book keeping, economic planning, and management
  - Public health/health promotion
  - Quality management
  - Epidemiology of drug use (pharmaco-epidemiology)
  - Economics of drug use (pharmaco-economics)
  - History of pharmacy
7. Generic competences (GENERIC)
  - General knowledge
  - Academic literacy
  - Languages
  - First aid
  - Communication
  - Management
  - Practical skills

### 3.7. Impact of the Bologna Principles

Respondents were asked how the principles outlined in the Bologna declaration on degree comparability and student and staff mobility, cited above, affect PET in their HEI:

1. Easily readable and comparable degrees? Issue of a Diploma Supplement?
2. Courses divided into two main cycles: three-year B and two-year M?
3. Relevance of B degree to the European labor market
4. Possibility for students with a three-year B degree from an HEI other than pharmacy (natural sciences, chemistry…) and/or from another country to enroll into the pharmacy M
5. Use of the European system of credits (ECTS) [17] to promote student mobility and/or lifelong learning
6. Efforts made to remove obstacles to student and staff mobility (with language courses…)
7. Numbers of Erasmus exchange staff and students.
8. Involvement in European co-operative programs on quality assurance with attempts to develop comparable criteria and methodologies?
9. European dimensions in higher education regarding curriculum development, general inter-institutional co-operation and integrated programs of study, training and research

### 3.8. Impact of EC Directive

Questions were asked as to what extent HEIs followed the EC directive on the sectoral profession of pharmacy, especially concerning the five-year, ‘tunnel’ degree structure imposed by the directive that is in opposition to the Bologna recommendations.

Questions were asked on the three main elements of directive:

1. Course length
2. Course content
3. Traineeship

## 4. Conclusions

Country profiles for European PET will be published in ‘pharmacy’. This article serves as an introduction to these surveys and as a vade mecum for the reader of such articles. The information contained in the country profile articles is of a descriptive nature; it will serve as a tool for those wishing to do educational research on European PET and/or promote student and staff mobility.

Data for individual countries are compared with European averages already published [15]. For such comparisons we used a European linear regression estimation. Data were taken from the 25 EU members of the EHEA that had at least one pharmacy department. The calculation was as follows: estimations of numbers of pharmacies, etc. (X, dependent variable) were calculated from the linear regression equation with the country population as the independent (Y) variable. It was assumed in this calculation that when X = 0, then Y = 0. The reported number for the country was then expressed as a ratio of the estimated number. This will be illustrated by an example using community pharmacies in France. The global EU data for X = country population (in millions × 10^‒6^) and Y = number of community pharmacies, gave a slope of 298 ± 18 (*n* = 25 countries). Thus the EU linear regression estimation of the number of pharmacies in France = 64.7 million × 298 = 19,280. The reported number of pharmacies in France is 23,133, thus giving a ratio compared to the estimate of 23,133/19,280 = 1.20. France therefore has 1.2 times more pharmacies than to be expected from the EU linear regression estimation or EU ‘average’.

## Abbreviations Used in the PHARMINE Survey

B	Bachelor level (first three years study following secondary school). This may be followed by a number, e.g., B1 = first year of bachelor studies.
M	Master level (fourth and fifth years of study)
D	Doctoral (Ph.D.) level. This will start after five years of study at an HEI (three years B plus two years M)
E&T	Education and training
PET	Pharmacy education and training
HEI	Higher education institution
LLL	Lifelong learning
R&D	Research and development
S	Semester. This may be followed by a number, e.g., S1/B1 = first semester of the first bachelor year
